# Tumor-infiltrating immune cell profiles and changes associate with additional trastuzumab in preoperative chemotherapy for patients with HER2-positive gastric cancer

**DOI:** 10.1038/s41416-024-02835-z

**Published:** 2024-09-23

**Authors:** Cong Chen, Jing Han, Qifei He, Qian Yao, Xueying Wang, Zuofu Peng, Yu Sun, Jiafu Ji, Xiaofang Xing

**Affiliations:** 1 Key Laboratory of Carcinogenesis and Translational Research (Ministry of Education), Gastrointestinal Cancer Center, Peking University Cancer Hospital & Institute, 100142 Beijing, China; 2 Department of Orthopedics, Shenzhen Institute of Translational Medicine, The First Affiliated Hospital of Shenzhen University, Shenzhen Second People’s Hospital, Shenzhen, 518035 China; 3 Key Laboratory of Carcinogenesis and Translational Research (Ministry of Education), Department of Pathology, Peking University Cancer Hospital & Institute, 100142 Beijing, China; 4 Alpha X(Beijing) Biotech Co., Ltd., 102629 Beijing, China; 5 State Key Laboratory of Holistic Integrative Management of Gastrointestinal Cancers, Beijing Key Laboratory of Carcinogenesis and Translational Research, Department of Pathology, Peking University Cancer Hospital & Institute, 100142 Beijing, China; 6 State Key Laboratory of Holistic Integrative Management of Gastrointestinal Cancers, Beijing Key Laboratory of Carcinogenesis and Translational Research, Gastrointestinal Cancer Center, Peking University Cancer Hospital & Institute, 100142 Beijing, China

**Keywords:** Gastric cancer, Cancer microenvironment

## Abstract

**Background:**

HER2(+) gastric cancer (GC) can benefit from trastuzumab. However, the impact of additional trastuzumab in preoperative treatment on immune cells remains largely unknown.

**Methods:**

In cohort I, immune cells were detected by immunohistochemistry in 1321 patients. Then 88 HER2(+) patients received preoperative therapy were collected as cohort II. Immune cell profiles and changes were analyzed in paired pre- and post-operative specimens using multiple immunohistochemistry staining.

**Results:**

In the treatment-naive GC patients (*n* = 1002), CD3+ and CD8+ T cell infiltration was significantly lower in the HER2(+) GC patients together with higher FoxP3+ T cells compared with HER2(−). However, FoxP3+ T and CD20+ B cell infiltration was significantly higher in HER2(+) GC after neoadjuvant chemotherapy (*n* = 319). The trastuzumab-exposed group had higher CD8+ T and lower FoxP3+ T cell infiltration and CD8+ T cell was even more significant in responders. Additionally, tertiary lymphoid structure (TLS) density increased in invasion margin of residual tumors. Patients with lower TLS in the tumor core or lower FoxP3+ T cells had better overall survival in the trastuzumab-exposed group.

**Conclusion:**

Addition of trastuzumab modulates the immune microenvironment, suggesting the potential mechanism of the favorable outcome of anti-HER2 therapy and providing a theoretical rationale for the combinational immunotherapy in resectable HER2(+) GC patients.

## Background

Gastric cancer (GC) is the fifth most diagnosed cancer and the fourth most common cancer-related death worldwide [1]. Because the survival benefit of standard chemotherapy is not promising, molecular-targeted therapy for GC has gained attention [2, 3]. Human epidermal growth factor receptor 2 (HER2), a member of the epidermal growth factor receptor (EGFR) family, is commonly overexpressed or amplified in about 17.9% of GC cases [4]. The first targeted agent shown to improve the prognosis of this disease was the HER2 antibody trastuzumab. Trastuzumab for Gastric Cancer (ToGA) study showed that trastuzumab plus chemotherapy improved overall survival compared with chemotherapy alone in treating metastatic, unresectable HER2 positive (HER2(+)) GC [5]. This study also included trastuzumab in the first-line treatment for patients with advanced HER2(+) GC [6, 7]. Due to the benefits of neoadjuvant chemotherapy (NACT) for patients with advanced GC, there has been interest in adding trastuzumab to the perioperative treatment of HER2(+) locally advanced GC in the past decade. Although it has not yet become a first-line treatment option, there are some clinical trials are ongoing shown that adding trastuzumab to NACT results in a higher pathological complete response rate [8–10]. However, studying the underlying mechanisms of trastuzumab’s antitumor activity is insufficient to inform clinical decision-making.

The interaction between cancer cells and immune cells in the tumor microenvironment (TME) is crucial for tumor occurrence and development [11]. Increasing evidence indicates the critical role of trastuzumab in the TME [12, 13]. Chemotherapy can modulate the TME through various mechanisms [14]. Recently, the significance of tumor-infiltrating lymphocyte (TIL) in response to NACT has been reported [15, 16]. Multiplex immunohistochemistry (mIHC) detected multiple antigens simultaneously at single-cell resolution, allowing more accurate delineation of immune cells’ density and spatial pattern in the TME [17]. The technique was used to analyze the changes in immune cells in the TME before and after NACT [18, 19]. A study reported the composition and dynamics of CD8+ and FoxP3+ T cells during trastuzumab neoadjuvant treatment in HER2(+) breast cancer [20]. However, HER2(+) GC’s immune status remains controversial, especially after NACT. It is also unknown how the additional trastuzumab to preoperative chemotherapy affects the composition and localization of immune cells in the TME in HER2(+) GC.

Tertiary lymphoid structure (TLS) is an aggregate of ectopic lymphocytes, usually composed of B cells with T cells and other immune cells surrounding the B cell core [21]. The density, maturity, and distribution of TLS in the TME are of increasing interest [22, 23]. TLS is associated with a favorable prognosis and response to GC immune checkpoint blockade (ICB) therapy [24, 25]. Studies on TLS in HER2(+) GC, especially TLS after chemotherapy and trastuzumab treatment, are scarce.

Therefore, studying TILs in HER2(+) GC and during anti-HER2 therapy is critical. Our study aimed to investigate TILs in the TME of HER2(+) GC during NACT and the effect of additional trastuzumab in preoperative chemotherapy and changes in CD8+ T cells, FoxP3+ T cells, and TLS in the TME of HER2(+) GC patients after preoperative chemotherapy and trastuzumab treatment by using the mIHC technique. The results of this analysis may help design new strategies for improving systemic therapy for HER2(+) GC.

## Methods

### Study design and patients

Cohort I: In a previously published article by our team, we described a cohort that included 1416 GC patients who underwent surgery [19]. 95 patients with unknown HER2 status were excluded from the previous cohort. A total of 1321 patients were included in this cohort and divided into two groups according to whether they received NACT (319 patients received NACT and 1002 patients did not receive NACT). The neoadjuvant chemotherapy regimen is the same as previous studies [19]. Immunohistochemistry (IHC) HER2 3+ is classified as HER2(+). Each group was further divided into subgroups according to HER2 status (Supplementary Fig. S1).

Cohort II: 88 GC patients admitted to our hospital were selected from January 1, 2010, to December 31, 2021.

Inclusion criteria: 1) Patients with newly diagnosed GC that was confirmed by pathology with complete clinicopathological and follow-up data; 2) Patients with positive HER2 IHC 3+ or IHC 2+ and fluorescence in situ hybridization, classified as HER2(+) [7]; 3) Patients receiving preoperative chemotherapy before undergoing primary D2 lymph node dissection.

Exclusion criteria: 1) Diagnosed with stage I disease according to the 8th edition of the American Joint Committee on Cancer Staging; 2) Experienced perioperative death; 3) Received perioperative radiotherapy, targeted therapies other than trastuzumab, immunotherapy, or unknown/absent perioperative therapies; 4) Complicated by additional malignancies.

Patients who received trastuzumab and chemotherapy treatment before surgery were in the trastuzumab-exposed group and only chemotherapy was defined as a control group. For cohort I, patients in NACT cohort received the XELOX or SOX for two to four cycles and without trastuzumab before surgery; for CohortII, patients received the XELOX/SOX/FOLFOX/FLOT for two to eight cycles with or without trastuzumab for two to six cycles and the average time from the first preoperative chemotherapy cycle to surgery is 122.6 days.

The study was approved by the Peking University Cancer Hospital Ethics Committee (No. 2021 KT15) and met the requirements of the Declaration of Helsinki.

### Data collection

Clinicopathological and treatment information were extracted from the hospital’s patient database. The clinical TNM (cTNM) classification was treated as the prechemotherapy TNM stage. The pathological TNM (pTNM) classification was treated as the post-chemotherapy TNM stage. Two types of outcomes, pathological outcome, and survival outcome, were considered for the cohort. Pathologists evaluated resected specimens and reported pathological outcomes, including tumor regression grade (TRG) (0, 1, 2, or 3) [7], pTNM stage (0, I, II, III, or IV), and the degree of tumor downstaging (cTNM stage minus pTNM stage; continuous value). The survival outcome was defined from the date of treatment (surgery date for the non-NACT cohort, or the date of the first preoperative chemotherapy cycle for the NACT cohort) to the date of death, loss to follow-up, or the last follow-up occasion.

### Tissue microarray and immune cell analysis

In this study, IHC technology was used to stain tissue microarray. We included common immunomarker proteins, including CD3 (pan T cells), CD4 (helper T cells), FoxP3 (regulatory T cells), CD8 (cytotoxic t lymphocytes), CD20 (B cells), CD57 (natural killer cells), and CD68 (macrophages). The percentage of positive cells (positive cells/(positive cells + negative cells) *100) indicated the infiltration of immune cells. Detailed information and methods were described in our previous research [19, 26].

### Multiplex immunohistochemistry for immune markers and analysis

Tyramide signal amplification (TSA) multiplexed fluorescent immunohistochemistry (mIHC) staining was performed on pre- and post-preoperative GC samples to phenotype different tumor-infiltrating T-cell populations and TLS using the AlphaTSA mIHC Kit (AXT37100031, AlphaX Bio, Beijing, China). Panel 1 (CD8, FoxP3, PANCK) was designed for the endoscopic biopsy samples, and Panel 2 (CD8, FoxP3, CD20, CD23, PANCK) for the surgical resection samples (detailed information is presented in Supplementary Table S1). Sections (5 μm thick) were cut from the GC tissues’ formalin fixation and paraffin embedding (FFPE) blocks. The slides underwent deparaffinization, rehydration, and washing before boiling in EDTA buffer at pH 9.0 for epitope retrieval. Endogenous peroxidase inhibition was achieved using 3% hydrogen peroxide. Primary antibodies were incubated for 1 h at room temperature. Incubation with XTSA secondary antibody was performed for 10 min at 37 °C. The slides were then incubated with XTSA TSA fluorochromes diluted in an amplification buffer for 10 min at RT. Either microwave treatment with EDTA buffer stripped the primary and secondary antibody complexes at pH 9.0. TSA single-stain slides were counterstained with DAPI for 5 min and were enclosed in a Mounting Medium (IH0252, Leagene, Beijing, China). Multiplex TSA IHC was optimized by testing each antibody separately to determine optimal order, incubation time, and antibody dilution.

Whole tissue images were acquired using a Zeiss Axioscan 7 slide scanner (Carl Zeiss). The acquired images were analyzed using ZEN 3.3 software. The data were analyzed using the pathology analysis software Halo (3.4, Indica Labs, United States). Based on the quantitative statistics of single cells, the results included the division of tumor and stroma area, multiple data types statistics of single marker and multiple marker combinations, and the number of positive cells, positive rate, and density of positive cells.

### Statistical analysis

IBM SPSS 23.0 software was used for data analysis. Categorical variables were compared using the chi-square test, and Fisher’s exact test was performed with an expected number of less than 5. Continuous variables conforming to the normal distribution were compared by t-test, and the Wilcoxon rank sum test was used for intergroup comparisons for non-normal distribution. A paired samples t-test was used to compare the continuous variables in preoperative and post-preoperative samples from the same patient. KM survival curves were used to illustrate OS, and the log-rank test was performed to compare the survival rates between groups. A univariable COX regression model was used to analyze the relationship between variables and survival, and a hazard ratio (HR) and *P* value were used to determine the impact of variables on prognosis. Variables with a *P* value less than 0.05 and basic patient information such as age and gender were included in the multivariate COX regression model to determine independent prognostic factors. A *P*-value of less than 0.05 was considered statistically significant.

## Results

### The remodeling effect of preoperative chemotherapy on the TME in HER2(+) GC patients

**Fig. 1:**
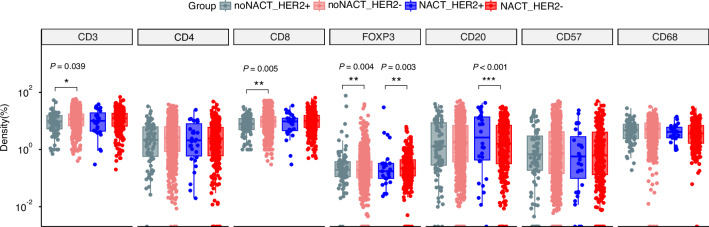
Comparison of immune cell infiltration of GC with different HER2 status in NACT or non-NACT group. Data are presented as boxplots, the boxplot center line indicates the median and the upper and lower box boundaries represent the 75th and 25th percentiles, respectively. *p < 0.05; **p < 0.01; ***p < 0.001. NACT neoadjuvant chemotherapy.

To understand the remodeling effects of NACT on the TME in HER2(+) GC patients, we divided the Cohort I into the NACT group (*n* = 319) and the non-NACT group (*n* = 1002). And the infiltration of CD3, CD4, CD8, FoxP3, CD57, CD20 and CD68 positive cells was compared, respectively. Baseline clinical characteristics of patients with different HER2 status in NACT or non-NACT group were shown in Supplementary Tables S2, 3. We found that in the non-NACT group, the infiltration of CD3+ (*P* = 0.039) and CD8+ cells (*P* = 0.005) in the HER2(+) group were significantly lower compared with that in the negative group, while the infiltration of FoxP3+ cells was significantly higher (*P* = 0.004), and the positive cells of CD20, CD68, and CD57 were not significantly changed. In the NACT group, FoxP3+ cells were still significantly higher (*P* = 0.003) and interestingly, CD20+ cells were significantly higher (*P* < 0.001) (Fig. 1). Next, univariate and multivariate COX survival analysis evaluated the relationship between clinicopathological factors and prognosis. We found that HER2 status was not associated with survival regardless of whether NACT was received (Supplementary Tables S4, 5).

The above results show that the immune status of HER2(+) GC patients was inhibited, and receiving NACT can help alleviate this state, and B cell infiltration was higher. Next, we focused on a retrospective analysis of our center’s HER2(+) patients with preoperative chemotherapy.

The characteristics of Cohort II are listed in Supplementary Table S6, of which 52 patients had paired preoperative endoscopic biopsy and intraoperative specimens (Supplementary Fig. S1). 80.7% were males, and the median age was 62 (range 25–78). All patients were diagnosed with locally advanced (89.8%) or metastatic (10.2%) HER2(+) GC or GEJ cancer and underwent gastrectomy. Patients were divided into two groups according to whether to add trastuzumab in the preoperative chemotherapy in Cohort II. Then, we compared the clinicopathological characteristics and treatment regimens of these two clusters of patients. As shown in Supplementary Table S7, no significant differences were observed between the groups.

### Pathological and survival outcomes of HER2(+) patients adding trastuzumab in preoperative chemotherapy

Survival analysis of Cohort II was used to determine the relationship between clinicopathological factors and OS. The median follow-up times for the trastuzumab-exposed and control groups were 22.7 and 53.3 months, respectively. We found that only tumor differentiation was an independent prognostic factor, but adding trastuzumab to preoperative chemotherapy did not have prognostic significance (Supplementary Table S8) (Supplementary Fig. S2). However, patients in the trastuzumab-exposed group had better pathological results, as reflected by tumor regression and downstaging (Table 1). Patients who received trastuzumab had lower TRG scores (*P* = 0.002) and higher degrees of tumor downstaging but not significantly (*P* = 0.088). The pTNM stage also tended to decrease but not significantly (*P* = 0.203). These results demonstrated that patients who added trastuzumab to preoperative chemotherapy had improved short-term pathological outcomes but did not have improved long-term survival outcomes.

**Table 1:** Outcomes of preoperative chemotherapy patients with or without trastuzumab.

Outcome	Trastuzumab	No-Trastuzumab	*P*-value
	(*n* = 50)	(*n* = 38)	
TRG			
0	4(8.0)	1(2.6)	0.002*
1	8(16.0)	5(13.2)	
2	26(52.0)	8(21.1)	
3	12(24.0)	24(63.2)	
pTNM stage			
0	4(8.0)	1(2.6)	0.203
I	7(14.0)	2(5.3)	
II	19(38.0)	17(44.7)	
III	15(30.0)	17(44.7)	
IV	5(10.0)	1(2.6)	
Tumor downstaging			
≤0	17(34.0)	16(42.1)	0.088
1	18(36.0)	18(47.4)	
≥2	15(30.0)	4(10.5)	

Significant value (*P* < 0.05) is in bold.

### Analysis strategy of multiplexed-imaging assay of immune cell infiltration in HER2(+) patients received preoperative chemotherapy

To further explore the changes in immune cell infiltration caused by trastuzumab, 52 patients with endoscopic specimens before treatment and surgical resection specimens were collected for mIHC staining (Supplementary Fig. S3a). The two panels (listed in Method) included a tumor-related marker (PANCK) for tumor and stroma recognition, two T-cell lineage markers (CD8 and FoxP3), and two TLS-related markers (CD20 and CD23) (Supplementary Fig. S3b, c). Cytotoxic T cells (CD8+) and regulatory T cells (FoxP3+) were selected as effector and suppressor functions in TME, respectively. TLS was identified by two pathologists using H&E staining tissue and mIHC. CD20+CD23− TLS was defined as immature TLS and CD20+CD23+TLS as mature TLS.

### Immune cell infiltration associated with trastuzumab treatment in HER2(+) patients received preoperative treatment

52 paired samples were analyzed after excluding samples with no tumor core and unrecognized fluorescence signal. These samples included 28 cases in the chemotherapy plus trastuzumab group (trastuzumab-exposed group) and 24 cases in the chemotherapy alone group (control group) (Supplementary Figs. S1, S4).

**Fig. 2:**
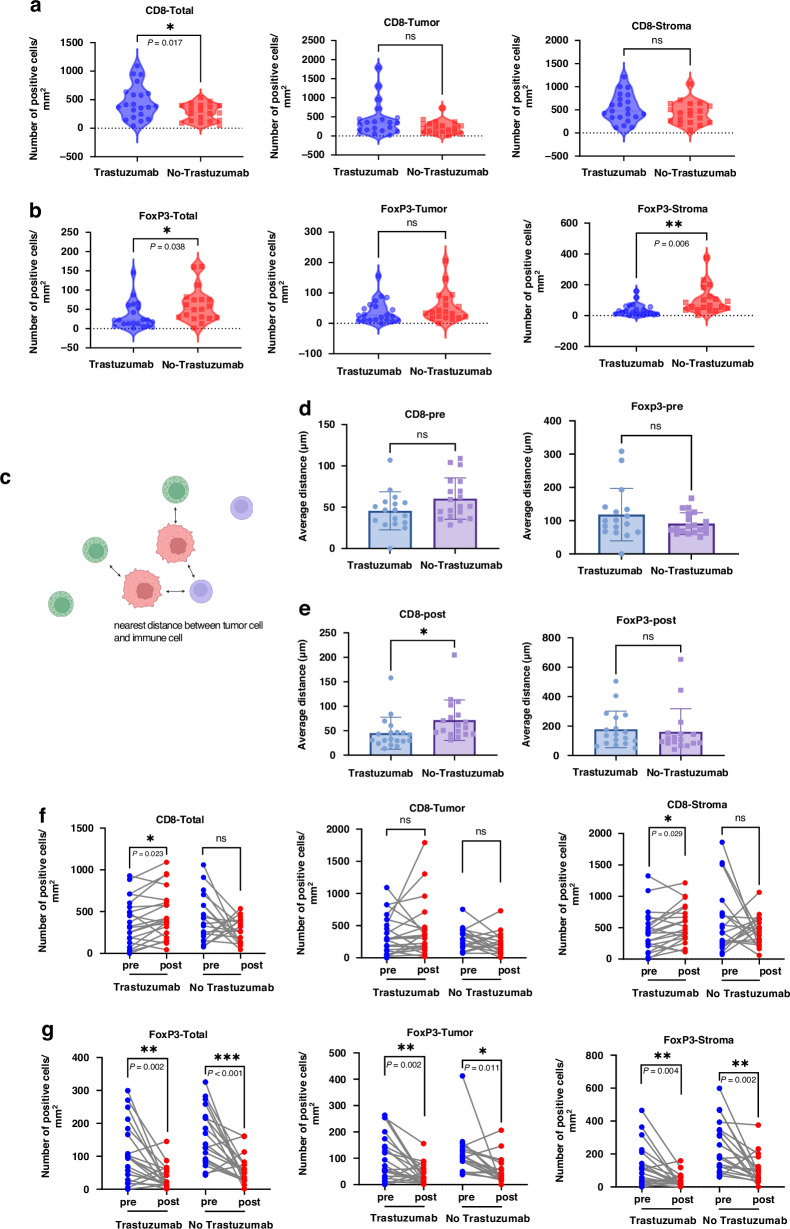
Immune cell infiltration associated with trastuzumab treatment in HER2(+) patients received preoperative treatment. **a** Comparison of CD8+ T cell density in total (left), tumor (middle) and stroma(right) after preoperative chemotherapy. **b** Comparison of FoxP3+ T cell density in total (left), tumor (middle) and stroma (right) after preoperative chemotherapy. **c** The schematic diagram of nearest distance calculation. **d** Comparison of average distance between CD8+ T cell and tumor cell. **e** Comparison of the average distance between FoxP3+ T cell and tumor cell. **f** Comparison of the alterations in total (left), tumor (middle) and stroma (right) CD8+ T cell in paired before and after treatment specimens with trastuzumab or not. **g** Comparison of the alterations in total (left), tumor (middle) and stroma (right) FoxP3+ T cell in paired before and after treatment specimens with trastuzumab or not. *p < 0.05; **p < 0.01; ***p < 0.001.

Before treatment, we compared immune cell infiltration in both groups and found no differences except for FoxP3+ T cells in the stroma (Supplementary Fig. S5). After preoperative treatment, total CD8+ T cells infiltrated in the trastuzumab-exposed group was significantly higher than in the control group (*P* = 0.017). We analyzed CD8+ T cell infiltration in tumor and stroma areas. In the trastuzumab-exposed group, CD8+ T cells tended to increase in both compartments, but the increase was insignificant (Fig. 2a). Meanwhile, we found that the total infiltration of FoxP3+ T cells was significantly lower in the trastuzumab-exposed group (*P* = 0.038) and significantly lower in the stroma area (*P* = 0.006) (Fig. 2b). Then, we took the tumor cells as the center and calculated the average distance of the nearest immune cells from the center (Fig. 2c). There was no significant difference in the distance between CD8+ and FoxP3+ T cells and tumor cells before preoperative treatment (Fig. 2d), and the average distance between CD8+ T cells and tumor cells was significantly shorter in the trastuzumab-exposed group (Fig. 2e).

Afterward, we compared the changes in CD8+ T cells and FoxP3+ T cells between the two patient groups in matched samples before and after preoperative treatment. We observed a significant increase in the total CD8+ T cells in the patients exposed to trastuzumab (*P* = 0.023). There was significant change in CD8+ T cells in the tumor area, mainly in the stromal area (*P* = 0.029), and no significant change in the control group (Fig. 2f). FoxP3+ T cells decreased significantly after preoperative treatment in both patient groups. In the tumor area, the decrease was more significant in the trastuzumab-exposed group (*P* = 0.002) (Fig. 2g).

### Immune cell infiltration relates to pathological tumor response in trastuzumab-exposed patients who received preoperative treatment

**Fig. 3:**
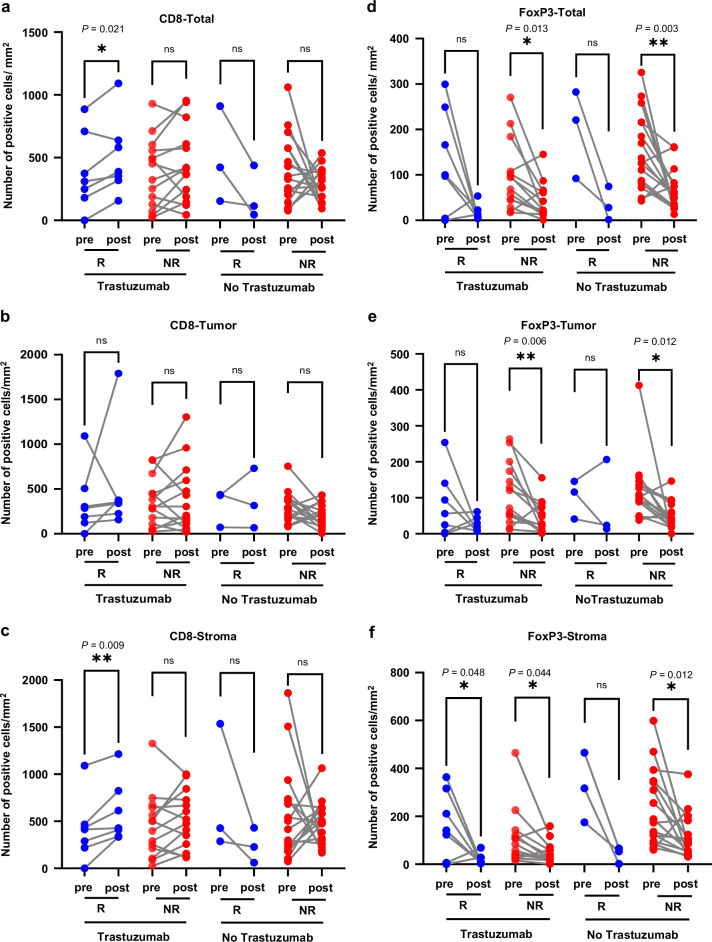
Immune cell infiltration relates to pathological tumor response in patients received preoperative treatment. Comparison of the alterations in CD8+ T cells total (**a**), tumor (**b**) and stroma (**c**) between the responder (TRG0-1) and non-responder (TRG2-3) with trastuzumab treatment or not. Comparison of the alterations in FoxP3+ T cells total (**d**), tumor (**e**), and stroma (**f**) between the responder (TRG0-1) and non-responder (TRG2-3) with trastuzumab treatment or not. *p < 0.05; **p < 0.01. R responder, NR non-responder.

To further compare the relationship between immune cell infiltration and pathological tumor response, we defined TRG grades 0-1 as responders and 2-3 as non-responders. There were 7 response cases and 14 non-response cases in the trastuzumab-exposed group and 3 response cases and 16 non-response cases in the control group, respectively. For patients in the trastuzumab-exposed group, the overall CD8+ T cell infiltration level significantly increased in patients who responded to the treatment (*P* = 0.021), particularly in the stroma area (*P* = 0.009). There was no significant change in CD8+ T cell infiltration in patients who did not respond to the treatment (Fig. 3a–c). As for FoxP3+ T cells, the level of infiltration in the stroma area decreased significantly in responders (*P* = 0.048), but there was no significant reduction in total and tumor area in responders. However, there was also a substantial decrease in infiltrating cells for non-responders (Fig. 3d–f). The results indicate that trastuzumab treatment can enhance CD8+ T cell infiltration, particularly in responders. CD8+ T cell infiltration in the stroma is more important for the responsiveness to additional trastuzumab treatment. Nevertheless, the treatment does not affect the infiltration of FoxP3+ T cells, and its correlation with treatment efficacy is not significant.

### Impact of distribution and maturation heterogeneities of tumor-specific TLS in HER2(+) patients who received preoperative treatment

**Fig. 4:**
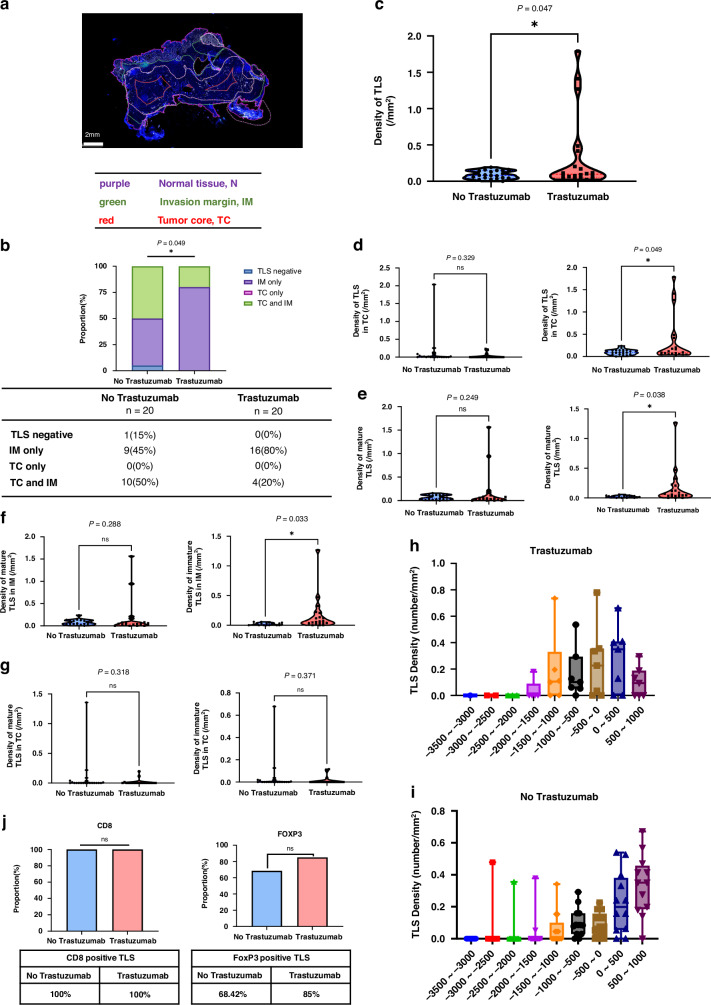
Impact of distribution and maturation heterogeneities of tumor-specific TLS in HER2(+) patients who received preoperative treatment. **a** Schematic diagram of regional division. **b** Differences in TLS density between preoperative treatment patients with or without trastuzumab. **c** Different distribution in TLS density between preoperative treatment patients with or without trastuzumab. **d** Differences in TLS distribution between preoperative treatment patients with or without trastuzumab. **e** Different maturity in TLS density between preoperative treatment patients with or without trastuzumab. **f** Differences in mature TLS density in IM between preoperative treatment patients with or without trastuzumab. **g** Differences in mature TLS density in TC between preoperative treatment patients with or without trastuzumab treatment patients with or without trastuzumab. **h** TLS distribution in preoperative treatment patients with trastuzumab. **i** TLS distribution in preoperative treatment patients without trastuzumab. **j** Differences in TLS status according to the infiltration of CD8+/FoxP3+ cells in preoperative chemotherapy patients with or without trastuzumab. *p < 0.05.

The T-cell-based immunotherapy strategy focuses on targeting TLS in the immune microenvironment, leading to increased interest in analyzing the status of TLS. We categorized the samples into tumor core (TC), invasion margin (IM), and adjacent normal tissue (N) for analysis (Fig. 4a). Only one sample in the control group lacked TLS, and no samples were exclusively distributed in TC. There was a higher proportion of TLSs found at IM in the trastuzumab-exposed group (80%) compared to the control group, where TLSs were distributed in TC (45%) (Fig. 4b). We found that the density of TLSs was higher in the trastuzumab-exposed group compared to the control group (*P* = 0.047) (Fig. 4c). Further analysis by different regions showed a significant increase in TLS density at IM in the trastuzumab-exposed group (*P* = 0.049), while no density changes in TC (Fig. 4d). Regarding the maturity of TLSs, we found a significantly higher density of immature TLSs in the trastuzumab-exposed group (*P* = 0.038) (Fig. 4e). We compared the density of TLSs with various maturity levels in different regions. We found a significant increase in immature TLSs at IM (*P* = 0.033). However, no difference was observed in mature TLSs (Fig. 4f). No difference was observed in TC (Fig. 4g). We set the tumor margin as 0μm, and observed the TLS distribution by zoning inward and outward at intervals of 500 μm. The TLSs in the trastuzumab treatment group tended to be distributed toward the TC (Fig. 4h, i) (Supplementary Fig. S6). Furthermore, the two groups had no significant differences regarding the CD8+ T cells and FoxP3 + T cells surrounding the TLSs (Fig. 4j).

### The low density of TLS in TC and infiltration of FoxP3+ T cells indicated a better prognosis in the trastuzumab-exposed group

Then survival analysis was performed to analyze the relationship between prognosis and immune cell infiltration. Multivariate COX survival analysis showed that FoxP3+ T cell levels before treatment were an independent factor (HR: 1.009; 95% CI: 1.002-1.016; *P* = 0.007) together with the pM stage. HER2(+) patients with a lower infiltration of FoxP3+ T cell had a better OS (Supplementary Table S9). Then, we analyzed the impact of pre- and post-preoperative CD8+ and FoxP3+ T cell density in two groups, and found that lower infiltration of FoxP3+ T cell has a better prognosis (HR: 1.019; 95% CI: 1.004–1.035; *P* = 0.014). Multivariate analysis indicated that FoxP3+ T cell was an independent prognostic factor (HR: 1.080; 95% CI: 1.001–1.165; *P* = 0.047). After analyzing the TLSs and prognosis, we found that the density, maturity, and distribution of TLSs did not significantly affect patient without trastuzumab prognosis. High TLS density in total and in IM indicated a better prognosis in the trastuzumab-exposed group but there was no statistical difference. However, we found that a lower density of TLS in TC indicated a better prognosis in the trastuzumab-exposed group (HR: 1.149; 95% CI: 1.028–1.285; *P* = 0.015) and was an independent prognostic factor in multivariate analysis (HR: 1.179; 95% CI: 1.035–1.343; *P* = 0.013) (Table 2).

**Table 2:** Univariate and multivariate COX analysis of the impact of CD8, FoxP3 and TLS expression in pre- and post-preoperative chemotherapy on OS in patients with or without trastuzumab.

			No Trastuzumab		Trastuzumab	
			HR (95% CI)	*P*-value	HR (95% CI)	*P*-value
CD8	Pre	Total	0.999 (0.997−1.002)	0.719	0.998 (0.994−1.002)	0.424
		Tumor	0.998 (0.992−1.003)	0.353	0.999 (0.995−1.003)	0.753
		Stroma	1.000 (0.998−1.001)	0.661	0.998 (0.994−1.001)	0.204
	Post	Total	1.000 (0.994−1.006)	0.844	0.998 (0.994−1.002)	0.358
		Tumor	1.001 (0.997−1.005)	0.673	0.998 (0.995−1.002)	0.386
		Stroma	0.998 (0.995−1.002)	0.354	0.998 (0.994−1.002)	0.319
FoxP3	Pre	Total	1.001 (0.992−1.010)	0.832	0.999 (0.990−1.009)	0.886
		Tumor	1.006 (0.998−1.014)	0.154	**1.019 (1.004−1.035)**	0.014*
		Tumor (Multi)^a^			**1.080 (1.001−1.165)**	0.047*
		Stroma	0.999 (0.994−1.004)	0.581	0.995 (0.984−1.005)	0.334
	Post	Total	0.986 (0.962−1.011)	0.263	0.991 (0.956−1.026)	0.597
		Tumor	0.999 (0.986−1.013)	0.941	1.007 (0.981−1.033)	0.602
		Stroma	0.991 (0.975−1.006)	0.248	0.985 (0.952−1.019)	0.396
TLS Density	Post	Total	1.146 (0.964−1.363)	0.123	0.992 (0.974−1.011)	0.411
		Mature	1.113 (0.943−1.314)	0.205	0.923 (0.781−1.091)	0.345
		Immature	1.384 (0.817−2.342)	0.227	1.003 (0.979−1.027)	0.814
		in TC	0.999 (0.973−1.026)	0.948	**1.149 (1.028−1.285)**	0.015*
		in TC(Multi)			**1.179 (1.035−1.343)**	0.013*
		in IM	1.114 (0.982−1.263)	0.094	0.992 (0.973−1.011)	0.394

Variables with *P* value less than 0.05 in univariate COX analysis and basic information of patients such as age and gender were included in the multivariate COX analysis.

Significant value (*P* < 0.05) is in bold.

^a^Multi, Multivariate COX analysis.

## Discussion

With the advent of immunotherapy, there has been a growing focus on immune cells in the TME. Several studies have demonstrated that NACT, when combined with surgical treatment, can lead to better curative resection rates, reduce the risk of micrometastasis, and prolong the overall survival of advanced GC patients [27, 28]. As a result, an increasing number of projects examined the changes in immune cells in the TME before and after NACT, providing a theoretical basis for combining immunotherapy with NACT. There are few studies on the impact of NACT on the TME for HER2(+) GC, let alone less research on the changes of immune-infiltrating cells in the TME before and after NACT with Trastuzumab in HER2(+) GC. Our study characterized the immune microenvironment of HER2(+) GC in NACT and non-NACT groups, compared the pathological and survival outcomes of patients receiving preoperative chemotherapy plus trastuzumab versus preoperative chemotherapy alone and, for the first time, analyzed the changes of immune-infiltrating cells in TME induced by trastuzumab using mIHC techniques.

Our analysis of IHC results showed that HER2(+) GC patients have fewer CD3 and CD8+ T cells, while FoxP3+ T cells increase, and have a relatively suppressive immune status, consistent with previous studies [29, 30]. Our study with a larger sample size is more convincing. In addition, there was no statistical difference in CD3 and CD8 in the NAC group, and CD20 increased, indicating that NAC relatively increased the infiltration of cytotoxic T cells, and B cells might play an important role.

Collecting paired tissue specimens before and after preoperative treatment in HER2(+) GC patients was challenging. However, we gathered paired samples from resectable locally advanced GC patients before and after NACT. Additionally, we obtained paired samples from patients who underwent successful conversion therapy. This allowed us to have a relatively larger cohort compared to previous studies. This study showed notable tumor regression and downstaging in the group that received trastuzumab but no difference in OS, which aligns with prior research [8–10, 31]. One reason for the lack of benefits from OS was the relatively small sample size. Another notable difference was the median follow-up time between the control group (53.3 months) and the trastuzumab-exposed group (22.7 months).

We found that the total infiltration level of CD8+ T cells increased, and the total infiltration of FoxP3+ T cells decreased in the preoperative chemotherapy plus trastuzumab group compared to the preoperative chemotherapy alone group. These implied that trastuzumab might promote the formation of an activated immune microenvironment. Previous studies have shown that HER2 signaling abnormalities could contribute to the evasion of the antitumor immune response. This may occur through potential mechanisms, such as TBK1 phosphorylation loss caused by HER2 amplification, weakened STING signaling, and subsequent interferon and antitumor immune response impairment. HER2 expression significantly reduces the infiltration of immune cells, including CD4+ and CD8+ T cells, in mouse melanoma [32, 33]. A single-cell RNA sequencing study on samples from 5 HER2(+) GC patients before treatment and 2 patients after two cycles of chemotherapy plus trastuzumab revealed that the cGAS-STING pathway was significantly activated compared to HER2(-) patients [34]. Trastuzumab might recruit additional CD8+ T cells and inhibit FoxP3+ Treg cells, which was also observed in our study.

In the examination of paired samples, surprisingly, the preoperative chemotherapy alone group of HER2(+) GC patients exhibited a decrease in CD8+ T cell infiltration levels before treatment. This observation deviated from prior findings, as earlier investigations have indicated that NACT may heighten the infiltration of CD8+ T cells within TME, implying that chemotherapy could intensify the expansion and activation of cytotoxic T cells within TME, thereby augmenting antitumor immunity. Given that HER2(+) individuals only comprise roughly 15% of GC patients, antecedent reports primarily centered on HER2(-) patients, whereas our cohort encompassed HER2(+) patients [16, 18]. For patients with HER2(+) GC, relying solely on preoperative chemotherapy may not be enough to boost CD8+ T cell numbers. On the other hand, trastuzumab use has been found to significantly increase the infiltration levels of CD8+ T cells, suggesting that immune activation may be necessary for HER2(+) GC patients. For FoxP3+ T cells, both groups showed a significant decrease in cell counts after preoperative treatment, and the addition of trastuzumab resulted in even lower infiltration levels of FoxP3+ T cells. These data support the notion that trastuzumab can induce immune activation.

Previous studies have shown that treatment with NACT significantly decreases the expression of stroma FoxP3+ T cells [19]. Our study showed that the presence of FoxP3+ T cells was not a reliable predictor of chemotherapy efficacy in HER2(+) GC, likely due to the small sample size. In our cohort, we found that patients who responded to treatment with the addition of trastuzumab showed a significant increase in CD8+ T cells after treatment, particularly in the stroma region rather than the tumor region. This indicated that increased CD8+ T cell infiltration in the stroma might predict the effectiveness of preoperative treatment and that the localization of CD8+ T cells might influence the immune response.

Our research for the first time revealed that trastuzumab could increase the presence of TLS in TME. The literature presented a decrease in the number of TLS and impaired TLS function after chemotherapy [35]. Therefore, trastuzumab treatment can partially counteract the impact of chemotherapy on TLS and enhance the maintenance of immune responses. It has been reported that ICB therapy can induce the formation of TLS in tumors [36]. The heterogeneity of GC tissue may introduce bias, particularly in endoscopic biopsy samples, which hinders accurate analysis of TLS. Therefore, we only focused on TLS after treatment. Trastuzumab exposure led to an increase in immature TLS infiltration, which was mainly distributed at the IM. We speculated that the addition of ICB therapy in HER2(+) GC can further develop immature TLS located at the IM, allowing them to exert their effects in the TC. TLS can help maintain T-cell activity and reduce T-cell exhaustion, benefiting immune responses. Previous studies have compared the infiltration of CD8+ T cells and FoxP3+ T cells into TLS [25, 37]. There were no significant differences in the proportion of both cell types in GC with trastuzumab treatment, suggesting it is a unique characteristic of GC.

As a retrospective study, it certainly has limitations-first, a limited number of preoperative and postoperative samples, resulting in limited statistical power. Larger sample sizes are needed to increase the reliability of our preliminary conclusions and determine the prognostic value. Second, we primarily focused on CD8+ T cells and FoxP3+ T cells. Investigations of NK cells, dendritic cells, and macrophages, as well as common immune markers and checkpoints expressions in GC, might be involved in the future. Third, the HER2(−) GC patients or those without preoperative treatment could be included as supplementary data for the following study to uncover the unique immune microenvironment characteristics of HER2(+) GC patients.

Our study has the most significant samples to describe the immune cell infiltration in HER2(+) GC patients by IHC. It is the initial investigation of matched CD8+ T cells, FoxP3+ T cells, and TLS in HER2(+) GC patients pre- and post-trastuzumab in the preoperative chemotherapy regimen. The results indicate that HER2(+) GC patients may benefit from preoperative chemotherapy, and adding trastuzumab significantly increases CD8+ cytotoxic T cells, reduces FoxP3+ Treg cells, and increases TLS. The recent KEYNOTE-811 trial showed that the addition of the anti-programmed death 1 (PD-1) antibody pembrolizumab to trastuzumab and chemotherapy significantly reduced tumor size, induced a complete response in some participants, and significantly increased the objective response rate [38]. Our findings suggested that trastuzumab had immunomodulatory effects and explained why PD-1 inhibitors combined with trastuzumab are beneficial. It implied the potential for a combination of preoperative chemotherapy, trastuzumab, and immunotherapy for HER2(+) GC in clinical practice.

## Supplementary information

Supplemental material

## Data Availability

The data are available from the corresponding author on reasonable request.

## References

[1] Sung H, Ferlay J, Siegel RL, Laversanne M, Soerjomataram I, Jemal A, et al. Global Cancer Statistics 2020: GLOBOCAN Estimates of Incidence and Mortality Worldwide for 36 Cancers in 185 Countries. CA Cancer J Clin. 2021;71:209–49. 33538338 10.3322/caac.21660

[2] Al-Batran SE, Homann N, Pauligk C, Goetze TO, Meiler J, Kasper S, et al. Perioperative chemotherapy with fluorouracil plus leucovorin, oxaliplatin, and docetaxel versus fluorouracil or capecitabine plus cisplatin and epirubicin for locally advanced, resectable gastric or gastro-oesophageal junction adenocarcinoma (FLOT4): a randomised, phase 2/3 trial. Lancet. 2019;393:1948–57. 30982686 10.1016/S0140-6736(18)32557-1

[3] Zhu Y, Zhu X, Wei X, Tang C, Zhang W. HER2-targeted therapies in gastric cancer. Biochim Biophys Acta Rev Cancer. 2021;1876:188549. 33894300 10.1016/j.bbcan.2021.188549

[4] Abrahao-Machado LF, Scapulatempo-Neto C. HER2 testing in gastric cancer: An update. World J Gastroenterol. 2016;22:4619–25. 27217694 10.3748/wjg.v22.i19.4619 PMC4870069

[5] Bang YJ, Van Cutsem E, Feyereislova A, Chung HC, Shen L, Sawaki A, et al. Trastuzumab in combination with chemotherapy versus chemotherapy alone for treatment of HER2-positive advanced gastric or gastro-oesophageal junction cancer (ToGA): a phase 3, open-label, randomised controlled trial. Lancet. 2010;376:687–97. 20728210 10.1016/S0140-6736(10)61121-X

[6] Japanese Gastric Cancer Association. Japanese Gastric Cancer Treatment Guidelines 2021 (6th edition). Gastric Cancer. 2023;26:1–25. 10.1007/s10120-022-01331-8 PMC9813208 36342574

[7] Ajani JA, D’Amico TA, Bentrem DJ, Chao J, Cooke D, Corvera C, et al. Gastric Cancer, Version 2.2022, NCCN Clinical Practice Guidelines in Oncology. J Natl Compr Canc Netw. 2022;20:167–92. 35130500 10.6004/jnccn.2022.0008

[8] Hofheinz RD, Hegewisch-Becker S, Kunzmann V, Thuss-Patience P, Fuchs M, Homann N, et al. Trastuzumab in combination with 5-fluorouracil, leucovorin, oxaliplatin and docetaxel as perioperative treatment for patients with human epidermal growth factor receptor 2-positive locally advanced esophagogastric adenocarcinoma: A phase II trial of the Arbeitsgemeinschaft Internistische Onkologie Gastric Cancer Study Group. Int J Cancer. 2021;149:1322–31. 34019698 10.1002/ijc.33696

[9] Hofheinz R-D, Merx K, Haag GM, Springfeld C, Ettrich T, Borchert K, et al. FLOT Versus FLOT/Trastuzumab/Pertuzumab Perioperative Therapy of Human Epidermal Growth Factor Receptor 2–Positive Resectable Esophagogastric Adenocarcinoma: A Randomized Phase II Trial of the AIO EGA Study Group. J Clin Oncol. 2022;40:3750–61. 35709415 10.1200/JCO.22.00380

[10] Wagner AD, Grabsch HI, Mauer M, Marreaud S, Caballero C, Thuss-Patience P, et al. EORTC-1203-GITCG - the “INNOVATION”-trial: Effect of chemotherapy alone versus chemotherapy plus trastuzumab, versus chemotherapy plus trastuzumab plus pertuzumab, in the perioperative treatment of HER2 positive, gastric and gastroesophageal junction adenocarcinoma on pathologic response rate: a randomized phase II-intergroup trial of the EORTC-Gastrointestinal Tract Cancer Group, Korean Cancer Study Group and Dutch Upper GI-Cancer group. BMC Cancer. 2019;19:494. 31126258 10.1186/s12885-019-5675-4 PMC6534855

[11] Hanahan D, Weinberg RA. Hallmarks of cancer: the next generation. Cell. 2011;144:646–74. 21376230 10.1016/j.cell.2011.02.013

[12] Park S, Jiang Z, Mortenson ED, Deng L, Radkevich-Brown O, Yang X, et al. The therapeutic effect of anti-HER2/neu antibody depends on both innate and adaptive immunity. Cancer Cell. 2010;18:160–70. 20708157 10.1016/j.ccr.2010.06.014 PMC2923645

[13] Varadan V, Gilmore H, Miskimen KL, Tuck D, Parsai S, Awadallah A, et al. Immune Signatures Following Single Dose Trastuzumab Predict Pathologic Response to PreoperativeTrastuzumab and Chemotherapy in HER2-Positive Early Breast Cancer. Clin Cancer Res. 2016;22:3249–59. 26842237 10.1158/1078-0432.CCR-15-2021 PMC5439498

[14] Pfirschke C, Engblom C, Rickelt S, Cortez-Retamozo V, Garris C, Pucci F, et al. Immunogenic Chemotherapy Sensitizes Tumors to Checkpoint Blockade Therapy. Immunity. 2016;44:343–54. 26872698 10.1016/j.immuni.2015.11.024 PMC4758865

[15] Zurlo IV, Schino M, Strippoli A, Calegari MA, Cocomazzi A, Cassano A, et al. Predictive value of NLR, TILs (CD4+/CD8+) and PD-L1 expression for prognosis and response to preoperative chemotherapy in gastric cancer. Cancer Immunol Immunother. 2022;71:45–55. 34009410 10.1007/s00262-021-02960-1 PMC8738448

[16] Christina Svensson M, Lindén A, Nygaard J, Borg D, Hedner C, Nodin B, et al. T cells, B cells, and PD-L1 expression in esophageal and gastric adenocarcinoma before and after neoadjuvant chemotherapy: relationship with histopathological response and survival. Oncoimmunology. 2021;10:1921443. 34104541 10.1080/2162402X.2021.1921443 PMC8158033

[17] Chen Y, Jia K, Sun Y, Zhang C, Li Y, Zhang L, et al. Predicting response to immunotherapy in gastric cancer via multi-dimensional analyses of the tumour immune microenvironment. Nat Commun. 2022;13:4851. 35982052 10.1038/s41467-022-32570-z PMC9388563

[18] Yu Y, Ma X, Zhang Y, Zhang Y, Ying J, Zhang W, et al. Changes in Expression of Multiple Checkpoint Molecules and Infiltration of Tumor Immune Cells after Neoadjuvant Chemotherapy in Gastric Cancer. J Cancer. 2019;10:2754–63. 31258783 10.7150/jca.31755 PMC6584940

[19] Xing X, Shi J, Jia Y, Dou Y, Li Z, Dong B, et al. Effect of neoadjuvant chemotherapy on the immune microenvironment in gastric cancer as determined by multiplex immunofluorescence and T cell receptor repertoire analysis. J Immunother cancer. 2022;10:e003984. 35361730 10.1136/jitc-2021-003984 PMC8971786

[20] Griguolo G, Serna G, Pascual T, Fasani R, Guardia X, Chic N, et al. Immune microenvironment characterisation and dynamics during anti-HER2-based neoadjuvant treatment in HER2-positive breast cancer. NPJ Precis Oncol. 2021;5:23. 33742063 10.1038/s41698-021-00163-6 PMC7979716

[21] Sautès-Fridman C, Petitprez F, Calderaro J, Fridman WH. Tertiary lymphoid structures in the era of cancer immunotherapy. Nat Rev Cancer. 2019;19:307–25. 31092904 10.1038/s41568-019-0144-6

[22] Petitprez F, de Reyniès A, Keung EZ, Chen TW, Sun CM, Calderaro J, et al. B cells are associated with survival and immunotherapy response in sarcoma. Nature. 2020;577:556–60. 31942077 10.1038/s41586-019-1906-8

[23] Werner F, Wagner C, Simon M, Glatz K, Mertz KD, Läubli H, et al. A Standardized Analysis of Tertiary Lymphoid Structures in Human Melanoma: Disease Progression- and Tumor Site-Associated Changes With Germinal Center Alteration. Front Immunol. 2021;12:675146. 34248957 10.3389/fimmu.2021.675146 PMC8264652

[24] Jia L, Wang T, Zhao Y, Zhang S, Ba T, Kuai X, et al. Single-cell profiling of infiltrating B cells and tertiary lymphoid structures in the TME of gastric adenocarcinomas. Oncoimmunology. 2021;10:1969767. 34513317 10.1080/2162402X.2021.1969767 PMC8425751

[25] Jiang Q, Tian C, Wu H, Min L, Chen H, Chen L, et al. Tertiary lymphoid structure patterns predicted anti-PD1 therapeutic responses in gastric cancer. Chin J Cancer Res. 2022;34:365–82. 36199531 10.21147/j.issn.1000-9604.2022.04.05 PMC9468020

[26] Xing X, Jia S, Leng Y, Wang Q, Li Z, Dong B, et al. An integrated classifier improves prognostic accuracy in non-metastatic gastric cancer. Oncoimmunology. 2020;9:1792038. 32939321 10.1080/2162402X.2020.1792038 PMC7470183

[27] Cunningham D, Allum WH, Stenning SP, Thompson JN, Van de Velde CJ, Nicolson M, et al. Perioperative chemotherapy versus surgery alone for resectable gastroesophageal cancer. N Engl J Med. 2006;355:11–20. 16822992 10.1056/NEJMoa055531

[28] Jiang L, Yang KH, Guan QL, Chen Y, Zhao P, Tian JH. Survival benefit of neoadjuvant chemotherapy for resectable cancer of the gastric and gastroesophageal junction: a meta-analysis. J Clin Gastroenterol. 2015;49:387–94. 25144898 10.1097/MCG.0000000000000212

[29] Cen S, Xu H, Liu Z, Zhao R, Pan H, Han W. Immune microenvironment characteristics and their implications for immune checkpoint inhibitor efficacy in HER2-overexpressing gastric cancer. Clin Exp Immunol. 2022;207:318–28. 35553632 10.1093/cei/uxac007 PMC9113110

[30] Fukai S, Nakajima S, Saito M, Saito K, Kase K, Nakano H, et al. Down-regulation of stimulator of interferon genes (STING) expression and CD8(+) T-cell infiltration depending on HER2 heterogeneity in HER2-positive gastric cancer. Gastric Cancer. 2023;26:878–90. 37542528 10.1007/s10120-023-01417-x

[31] He Q, Chen J, Zhou K, Jin C, Wang A, Ji K, et al. Effect of Additional Trastuzumab in Neoadjuvant and Adjuvant Treatment for Patients with Resectable HER2-Positive Gastric Cancer. Ann Surg Oncol. 2021;28:4413–22. 33393029 10.1245/s10434-020-09405-6

[32] Kumagai S, Koyama S, Nishikawa H. Antitumour immunity regulated by aberrant ERBB family signalling. Nat Rev Cancer. 2021;21:181–97. 33462501 10.1038/s41568-020-00322-0

[33] Wu S, Zhang Q, Zhang F, Meng F, Liu S, Zhou R, et al. HER2 recruits AKT1 to disrupt STING signalling and suppress antiviral defence and antitumour immunity. Nat Cell Biol. 2019;21:1027–40. 31332347 10.1038/s41556-019-0352-z

[34] Kim R, An M, Lee H, Mehta A, Heo YJ, Kim KM, et al. Early Tumor-Immune Microenvironmental Remodeling and Response to First-Line Fluoropyrimidine and Platinum Chemotherapy in Advanced Gastric Cancer. Cancer Discov. 2022;12:984–1001. 34933901 10.1158/2159-8290.CD-21-0888 PMC9387589

[35] Siliņa K, Soltermann A, Attar FM, Casanova R, Uckeley ZM, Thut H, et al. Germinal Centers Determine the Prognostic Relevance of Tertiary Lymphoid Structures and Are Impaired by Corticosteroids in Lung Squamous Cell Carcinoma. Cancer Res. 2018;78:1308–20. 29279354 10.1158/0008-5472.CAN-17-1987

[36] Allen E, Jabouille A, Rivera LB, Lodewijckx I, Missiaen R, Steri V, et al. Combined antiangiogenic and anti-PD-L1 therapy stimulates tumor immunity through HEV formation. Sci Transl Med. 2017;9:eaak9679. 28404866 10.1126/scitranslmed.aak9679 PMC5554432

[37] Masuda T, Tanaka N, Takamatsu K, Hakozaki K, Takahashi R, Anno T, et al. Unique characteristics of tertiary lymphoid structures in kidney clear cell carcinoma: prognostic outcome and comparison with bladder cancer. J Immunother cancer. 2022;10:e003883. 35314433 10.1136/jitc-2021-003883 PMC8938705

[38] Janjigian YY, Kawazoe A, Yanez P, Li N, Lonardi S, Kolesnik O, et al. The KEYNOTE-811 trial of dual PD-1 and HER2 blockade in HER2-positive gastric cancer. Nature. 2021;600:727–30. 34912120 10.1038/s41586-021-04161-3 PMC8959470

