# Supplementary material for: Tumor-infiltrating immune cell profiles and changes associate with additional trastuzumab in preoperative chemotherapy for patients with HER2-positive gastric cancer
Source: Br J Cancer. 2024 Sep 23;131(9):1463–72. doi: 10.1038/s41416-024-02835-z (PMC11519888; doi:10.1038/s41416-024-02835-z)
Supplement: Supplementary file 1 — Supplemental material [file 41416_2024_2835_MOESM1_ESM.docx]

Table S1. Primary antibodies used for mIHC

| **Antibody** | **Company** | **Catalog Number** | **Species** | **Dilution** |
| --- | --- | --- | --- | --- |
| CD8 | ZSGB-Bio | ZA-0508 | Rabbit | 1:100 |
| FoxP3 | Cell Signaling Technology | CST98377S | Rabbit | 1:100 |
| CD20 | Abcam | ab78237 | Rabbit | 1:100 |
| CD23 | ZSGB-Bio | ZA-0516 | Rabbit | 1:200 |
| PANCK | ZSGB-Bio | ZM-0069 | Mouse | 1:100 |

Table S2. Baseline characteristics of HER2(+) or HER2(-) patients with NACT in CohortⅠ

| **Clinical characteristics** | | **HER2 status** | | **P-value** |
| --- | --- | --- | --- | --- |
|  |  | **HER2(+) (n=31)** | **HER2(-) (n=288)** |  |
| **Gender** | Male | 25(80.7) | 225(78.1) | 0.746 |
|  | Female | 6(19.4) | 63(21.9) |  |
| **Age** |  | 63(55-66) | 59(52-66) | 0.532 |
| **Histology** | Ade | 31(100.0) | 261(90.6) | 0.365 |
|  | Ring | 0(0.0) | 19(6.6) |  |
| **Histologic differentiation** | Poor | 9(29.0) | 135(46.9) | 0.049 |
|  | Well or moderate | 20(64.5) | 128(44.4) |  |
| **Lauren Classification** | Diffused | 5(16.1) | 62(21.5) | 0.391 |
|  | Intestinal | 23(74.2) | 173(60.1) |  |
|  | Mixed | 1(3.23) | 32(11.1) |  |
| **Vascular Invasion** | No | 19(61.3) | 171(59.4) | 0.981 |
|  | Yes | 12(38.7) | 109(37.9) |  |
| **T stage** | T1 | 1(3.2) | 8(2.8) | 0.376 |
|  | T2 | 7(22.6) | 38(13.2) |  |
|  | T3 | 20(64.5) | 184(63.9) |  |
|  | T4 | 3(9.7) | 55(19.1) |  |
| **N stage** | N0 | 9(29.0) | 75(26.0) | 0.080 |
|  | N1 | 1(3.2) | 61(21.2) |  |
|  | N2 | 5(16.3) | 56(19.4) |  |
|  | N3 | 14(45.2) | 90(31.3) |  |
| **M stage** | M0 | 22(71.0) | 238(82.6) | 0.159 |
|  | M1 | 7(22.6) | 37(12.9) |  |
| **pTNM stage** | I | 3(9.7) | 26(9.0) | 0.447 |
|  | II | 8(25.8) | 95(33.3) |  |
|  | III | 11(35.5) | 116(40.3) |  |
|  | Ⅳ | 7(22.6) | 37(12.9) |  |

Table S3. Baseline characteristics of HER2(+) or HER2(-) patients without NACT in CohortⅠ

| **Clinical characteristics** | | **HER2 status** | | **P-value** |
| --- | --- | --- | --- | --- |
|  |  | **HER2(+) (n=88)** | **HER2(-) (n=914)** |  |
| **Gender** | Male | 65(73.9) | 660(72.2) | 0.74 |
|  | Female | 23(26.1) | 254(27.8) |  |
| **Age** |  | 61(52-69) | 60(52-69) | 0.624 |
| **Histology** | Ade | 80(90.9) | 815(89.2) | 0.143 |
|  | Ring | 3(3.4) | 78(8.5) |  |
| **Histologic differentiation** | Poor | 24(27.3) | 444(48.6) | <0.001 |
|  | Well or moderate | 62(70.5) | 420(46.0) |  |
| **Lauren Classification** | Diffused | 7(8.0) | 236(25.8) | <0.001 |
|  | Intestinal | 70(79.6) | 486(53.2) |  |
|  | Mixed | 7(8.0) | 178(19.5) |  |
| **Vascular Invasion** | No | 44(50.0) | 408(44.6) | 0.292 |
|  | Yes | 42(47.7) | 494(54.1) |  |
| **T stage** | T1 | 10(11.4) | 54(5.9) | 0.166 |
|  | T2 | 12(13.6) | 107(11.7) |  |
|  | T3 | 49(55.7) | 592(64.8) |  |
|  | T4 | 16(18.2) | 161(17.7) |  |
| **N stage** | N0 | 27(30.7) | 226(24.7) | 0.419 |
|  | N1 | 16(18.2) | 143(15.7) |  |
|  | N2 | 12(13.6) | 174(19.0) |  |
|  | N3 | 33(37.5) | 366(40.0) |  |
| **M stage** | M0 | 78(88.6) | 825(90.3) | 0.958 |
|  | M1 | 6(6.8) | 62(6.8) |  |
| **pTNM stage** | I | 14(15.9) | 102(11.2) | 0.265 |
|  | II | 28(31.8) | 251(27.5) |  |
|  | III | 36(40.9) | 472(51.6) |  |
|  | Ⅳ | 6(6.8) | 62(6.8) |  |

Table S4. Univariate and multivariate survival analysis in patients with NACT in CohortⅠ

| **Characteristics** | **Univariate** |  | **Multivariate** |  |
| --- | --- | --- | --- | --- |
|  | **HR (95% CI)** | ***P* value** | **HR (95% CI)** | ***P* value** |
| **Gender** |  |  |  |  |
| Famale | 1 |  | 1 |  |
| Male | 0.782(0.554-1.105) | 0.291 | 0.811(0.503-1.307) | 0.389 |
| **Age** | 1.004(0.990-1.018) | 0.593 | 0.997(0.980-1.014) | 0.698 |
| **Histology** |  |  |  |  |
| Ade | 1 |  |  |  |
| SRCC | 1.505(0.872-2.599) | 0.142 |  |  |
| **Differentiation** |  |  |  |  |
| Poor | 1 |  | 1 |  |
| Well/Moderate | 0.594(0.438-0.806) | **<0.001** | 0.751(0.480-1.176) | 0.219 |
| **Tumor**  **diameter** |  |  |  |  |
| <5cm | 1 |  | 1 |  |
| >=5cm | 2.050(1.512-2.779) | **<0.001** | 1.202(0.816-1.771) | 0.353 |
| **Vascular**  **invasion** |  |  |  |  |
| Negative | 1 |  | 1 |  |
| Positive | 2.785(2.066-3.755) | **<0.001** | 1.217(0.831-1.783) | 0.312 |
| **Lauren**  **classification** |  |  |  |  |
| Diffused | 1 |  | 1 |  |
| Intestinal | 0.662(0.466-0.939) | **0.021** | 0.726(0.427-1.237) | 0.239 |
| Mixed | 1.195(0.728-1.961 | 0.482 | 0.902(0.466-1.746) | 0.760 |
| **pTNM stage** |  |  |  |  |
| Ⅰ | 1 |  | 1 |  |
| Ⅱ | 4.861(1.166-20.259) | **0.03** | 4.000(0.524-30.594) | 0.181 |
| Ⅲ | 19.141(4.711-77.764) | **<0.001** | 13.182(1.739-99.890) | 0.013 |
| Ⅳ | 30.777(7.413-127.783) | **<0.001** | 19.770(2.558-152.829) | **0.004** |
| **NAC response** |  |  |  |  |
| non-responders | 1 |  | 1 |  |
| responders | 0.456(0.248-0.839) | **0.012** | 1.238(0.478-3.211） | 0.660 |
| **HER2 status** |  |  |  |  |
| Negative | 1 |  |  |  |
| Positive | 1.285(0.807-2.046) | 0.291 |  |  |
| **CD4** | 0.983(0.957-1.011) | 0.232 |  |  |
| **CD20** | 0.996(0.975-1.018) | 0.714 |  |  |
| **CD57** | 1.014(0.993-1.036) | 0.202 |  |  |
| **FoxP3** | 1.013(0.920-1.115) | 0.791 |  |  |
| **CD68** | 1.003(0.970-1.037) | 0.849 |  |  |
| **CD3** | 0.979(0.963-0.995) | **0.011** | 1.006(0.979-1.034) | 0.651 |
| **CD8** | 0.971(0.953-0.990) | **0.003** | 0.968(0.938-1.000) | 0.052 |

Table S5. Univariate and multivariate survival analysis in patients without NACT in CohortⅠ

| **Characteristics** | **Univariate** |  | **Multivariate** |  |
| --- | --- | --- | --- | --- |
|  | **HR (95% CI)** | ***P* value** | **HR (95% CI)** | ***P* value** |
| **Gender** |  |  |  |  |
| Famale | 1 |  | 1 |  |
| Male | 1.084(0.884-1.330) | 0.439 | 1.029(0.814-1.301) | 0.810 |
| **Age** | 1.018(0.990-1.026) | **<0.001** | 1.022(1.013-1.032) | **<0.001** |
| **Histology** |  |  |  |  |
| Ade | 1 |  |  |  |
| SRCC | 1.156(0.883-1.578) | 0.360 |  |  |
| **Differentiation** |  |  |  |  |
| Poor | 1 |  |  |  |
| Well/Moderate | 0.848(0.703-1.022) | 0.084 |  |  |
| **Tumor diameter** |  |  |  |  |
| <5cm | 1 |  | 1 |  |
| >=5cm | 2.002(1.660-2.415) | **<0.001** | 1.240(1.001-1.537) | **0.049** |
| **Vascular**  **invasion** |  |  |  |  |
| Negative | 1 |  | 1 |  |
| Positive | 2.140(1.764-2.597) | **<0.001** | 1.350(1.079-1.688) | **0.009** |
| **Lauren**  **classification** |  |  |  |  |
| Diffused | 1 |  | 1 |  |
| Intestinal | 0.722(0.583-0.891) | **0.003** | 0.643(0.481-0.859) | **0.003** |
| Mixed | 0.897(0.688-1.169) | 0.421 | 0.831(0.613-1.127) | 0.234 |
| **pTNM stage** |  |  |  |  |
| Ⅰ | 1 |  | 1 |  |
| Ⅱ | 2.900(1.648-5.102) | **<0.001** | 3.100(1.545-6.220) | **0.001** |
| Ⅲ | 7.388(4.320-12.633) | **<0.001** | 6.696(3.382-13.256) | **<0.001** |
| Ⅳ | 23.135(12.888-41.530) | **<0.001** | 21.394(10.249-44.649) | **<0.001** |
| **HER2 status** |  |  |  |  |
| Negative | 1 |  |  |  |
| Positive | 0.725(0.506-1.039) | 0.080 |  |  |
| **CD4** | 0.976(0.959-0.994) | **0.011** | 0.987(0.967-1.007) | 0.203 |
| **CD20** | 0.974(0.959-0.990) | **0.001** | 1.001(0.983-1.019) | 0.904 |
| **CD57** | 0.995(0.981-1.009) | 0.455 |  |  |
| **FoxP3** | 1.014(0.920-1.115) | 0.274 |  |  |
| **CD68** | 1.003(0.989-1.040) | 0.580 |  |  |
| **CD3** | 0.973(0.962-0.984) | **<0.001** | 1.006(0.979-1.034) | **0.026** |
| **CD8** | 0.971(0.957-0.984) | **<0.001** | 0.992(0.974-1.012) | 0.459 |

Table S6. Baseline clinical characteristics of HER2+ patients in Cohort Ⅱ

| **Clinical characteristics** |  | **Number of cases (%)** |
| --- | --- | --- |
| **Gender** | Male | 71(80.7) |
|  | Female | 17(19.3) |
| **Median age(range)** |  | 62(25-78) |
| **Charlson–Deyo comorbidity score** | 0 | 51(58.0) |
|  | 1 | 29(33.0) |
|  | >=2 | 8(9.1) |
| **Location** | EGJ | 43(48.9) |
|  | Gastric | 45(51.1) |
|  | Poor | 48(54.5) |
| **Histologic differentiation** | Well or moderate | 37(42.0) |
|  | N/A | 3(3.4) |
| **Type of surgery** | Total gastrectomy | 50(56.8) |
|  | Subtotal gastrectomy | 38(43.2) |
|  | I | 0(0) |
| **cTNM stage** | II | 2(2.3) |
|  | III | 70(79.5) |
|  | Ⅳ | 16(18.2) |
|  | SOX | 48(54.5) |
|  | XELOX | 20(22.7) |
| **Chemotherapy regimen** | FOLFOX | 3(3.4) |
|  | FLOT | 16(18.2) |
|  | Other | 1(1.1) |

Table S7. Baseline characteristics of the preoperative chemotherapy patients with or without trastuzumab in Cohort Ⅱ

| **Characteristics** |  | **Trastuzumab**  **(n=50)** | **No-Trastuzumab**  **(n=38)** | ***P*-value** |
| --- | --- | --- | --- | --- |
| **Gender** | Male | 38(76.0) | 33(86.8) | 0.202 |
|  | Female | 12(24.0) | 5(13.2) |  |
| **Age** |  | 63.0(25-78) | 59.5(43-72) | 0.221 |
| **Charlson–Deyo comorbidity score** | 0 | 30(60.0) | 21(55.3) |  |
|  | 1 | 16(32.0) | 13(34.2) | 0.875 |
|  | >=2 | 4(8.0) | 4(10.5) |  |
| **Location** | EGJ | 23(46.0) | 20(52.6) | 0.538 |
|  | Gastric | 27(54.0) | 18(47.4) |  |
| **Histologic differentiation** | Poor | 32(64.0) | 16(42.1) |  |
|  | Well or moderate | 16(32.0) | 21(55.3) | 0.091 |
|  | N/A | 2(4.0) | 1(2.6) |  |
|  | Total gastrectomy | 30(60.0) | 20(52.6) |  |
| **Type of surgery** | Subtotal  gastrectomy | 20(40.0) | 18(47.4) | 0.489 |
|  | I | 0(0.0) | 0(0.0) |  |
| **cTNM stage** | II | 1(2.0) | 1(2.6) | 0.093 |
|  | III | 36(72.0) | 34(89.5) |  |
|  | Ⅳ | 13(26.0) | 3(7.9) |  |
|  | SOX | 29(58.0) | 19(50.0) |  |
| **Chemotherapy regimen** | XELOX | 12(24.0) | 8(21.1) |  |
|  | FOLFOX | 1(6.0) | 2(5.3) |  |
|  | FLOT/POS | 8(16.0) | 8(21.1) |  |
|  | Others | 0(0.0) | 1(2.6) | 0.979 |

Table S8. Univariate and multivariate survival analysis in patients in Cohort Ⅱ

| **Characteristics** | **Univariate** |  | **Multivariate** |  |
| --- | --- | --- | --- | --- |
|  | **HR (95% CI)** | ***P*-value** | **HR (95% CI)** | ***P*-value** |
| **Gender** |  |  |  |  |
| Male | 1 |  | 1 |  |
| Female | 0.69 (0.26-1.84) | 0.455 | 0.71 (0.24-2.06) | 0.527 |
| **Age** |  |  |  |  |
| <=60 | 1 |  | 1 |  |
| >60 | 0.90 (0.42-1.92) | 0.776 | 1.21 (0.50-2.96) | 0.677 |
| **Charlson–Deyo comorbidity score** |  |  |  |  |
| 0 | 1 |  |  |  |
| 1 | 0.72 (0.31-1.67) | 0.447 |  |  |
| >=2 | 0.27 (0.04-2.06) | 0.208 |  |  |
| **Location** |  |  |  |  |
| EGJ | 1 |  |  |  |
| Gastric | 1.57 (0.73-3.39) | 0.25 |  |  |
| **Histologic differentiation** |  |  |  |  |
| Poor | 1 |  | 1 |  |
| Well or moderate | 0.34 (0.13-0.84) | 0.02 | 0.29 (0.11-0.77) | 0.013 |
| **surgical type** |  |  |  |  |
| Total gastrectomy | 1 |  |  |  |
| Subtotal gastrectomy | 0.61 (0.27-1.36) | 0.224 |  |  |
| **cT stage** |  |  |  |  |
| T3 | 1 |  |  |  |
| T4 | 0.52 (0.22-1.22) | 0.13 |  |  |
| **cN stage** |  |  |  |  |
| N0 | 1 |  |  |  |
| N1 | 1.95 (0.23-16.89) | 0.544 |  |  |
| N2 | 0.86 (0.11-6.74) | 0.886 |  |  |
| N3 | 2.93 (0.35-24.34) | 0.319 |  |  |
| **cM stage** |  |  |  |  |
| M0 | 1 |  |  |  |
| M1 | 2.46 (0.84-7.21) | 0.102 |  |  |
| **cTNM stage** |  |  |  |  |
| II | 1 |  |  |  |
| III | 1.21 (0.16-9.12) | 0.855 |  |  |
| Ⅳ | 1.49 (0.18-12.45) | 0.714 |  |  |
| **regimen** |  |  |  |  |
| SOX | 1 |  |  |  |
| XELOX | 0.37 (0.13-1.12) | 0.077 |  |  |
| FOLFOX | 1.39 (0.32-6.06) | 0.663 |  |  |
| FLOT/POS | 1.02 (0.34-3.07) | 0.978 |  |  |
| **Trastuzumab**  **treatment** |  |  |  |  |
| with | 1 |  | 1 |  |
| without | 0.84 (0.39-1.84) | 0.667 | 0.64 (0.28-1.48) | 0.297 |

Table S9. Univariate and multivariate survival analysis in 52


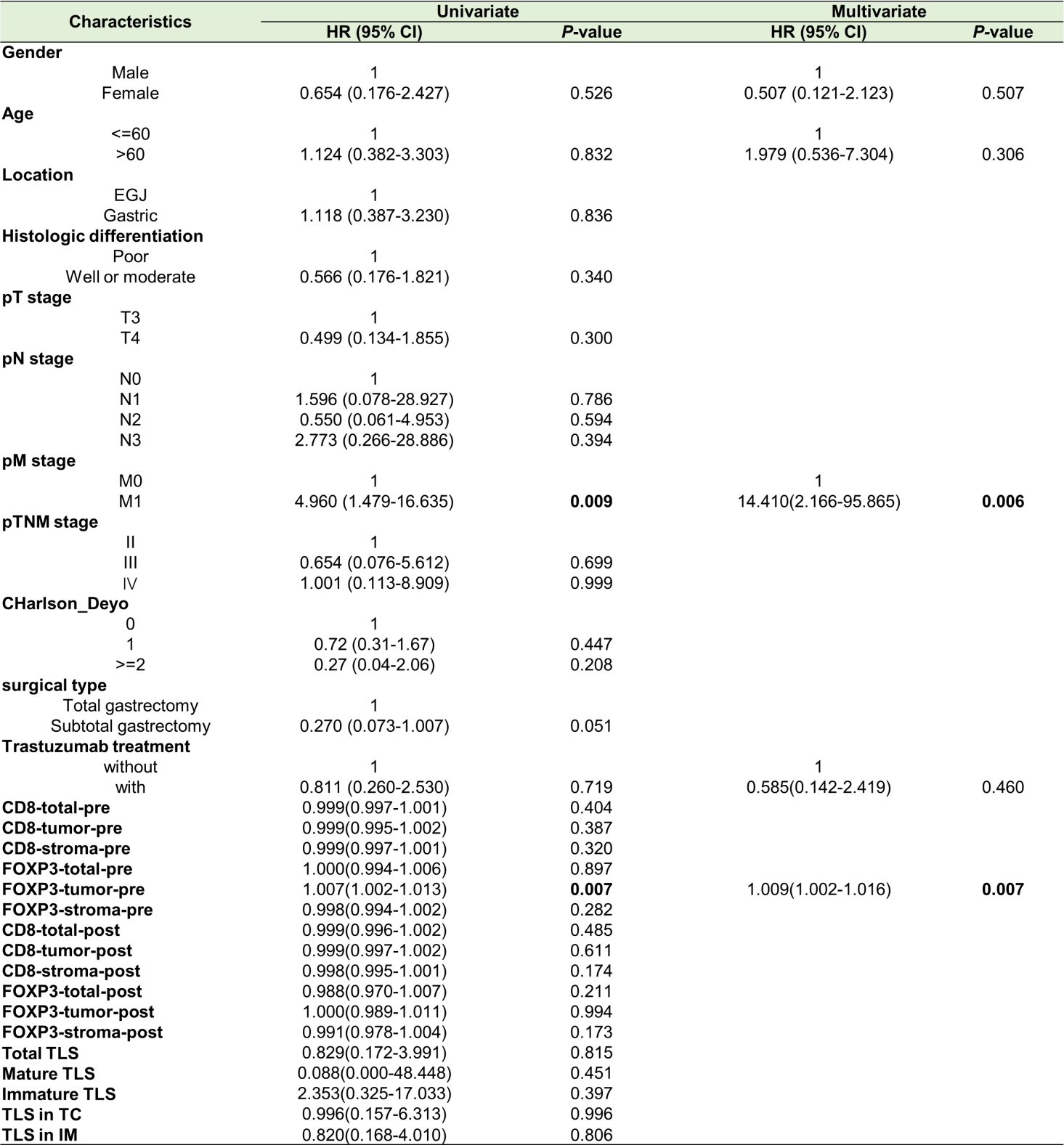
HER2(+) patients in Cohort Ⅱ

**cohort 2**


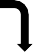


**Control group (n =38)**

**Patients with HER2+ GC underwent preoperative treatment and surgery**

**(n = 88)**

**Trastuzumab-exposed group (n = 50)**

**HER2(-) group (n =288)**

**Immunohistochemistry CD3/CD4/CD8/FoxP3/ CD20/CD57/CD68**

**Patients with NACT (n = 319)**

**HER2(+) group (n = 31)**

**Surgical sample**

**HER2(-) group (n =914 )**

**HER2(+) group (n = 88)**

**Patients without NACT (n = 1002)**

**cohort 1**

**Multiple immunohistochemistry Panel1:CD8/FOXP3/ PANCK**

**Panel2:CD8/FOXP3/ CD20/CD23/PANCK**

**Trastuzumab-exposed group (n = 28)**

Figure S1. The flow chat of the study.


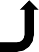


**Control group (n =24)**

**Surgical sample**

**biopsy sample**

**Patients with paired endoscopic biopsies and corresponding**

**surgical samples (n = 52)**


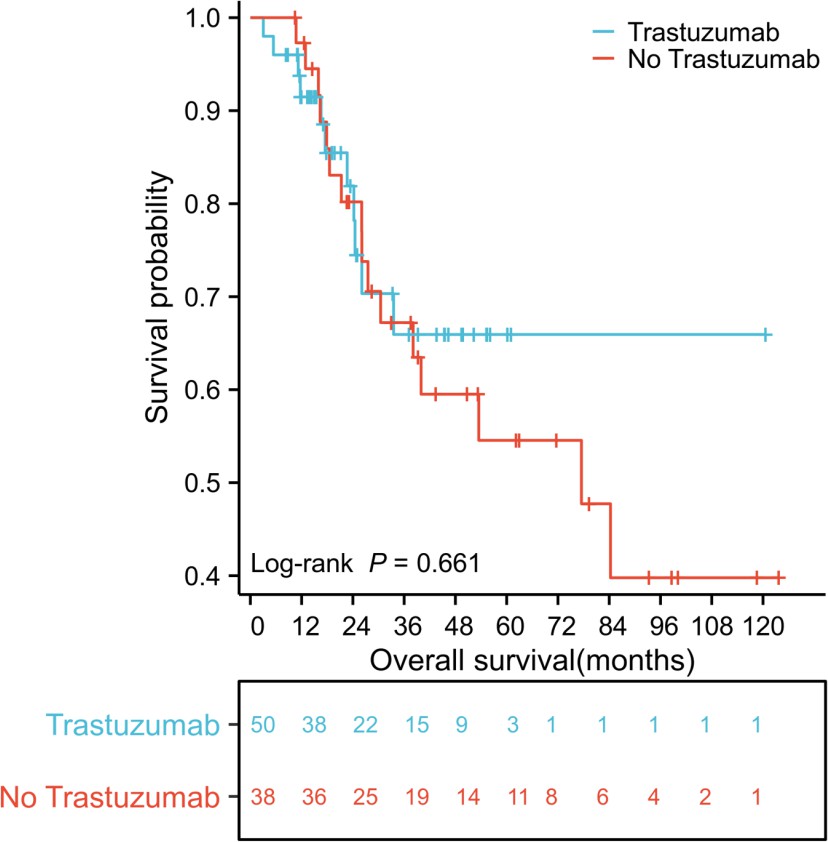


Figure S2. Kaplan–Meier survival curves of overall survival among HER2(+) GC patients with preoperative treatment. The *P* value was obtained from the log-rank test.

#
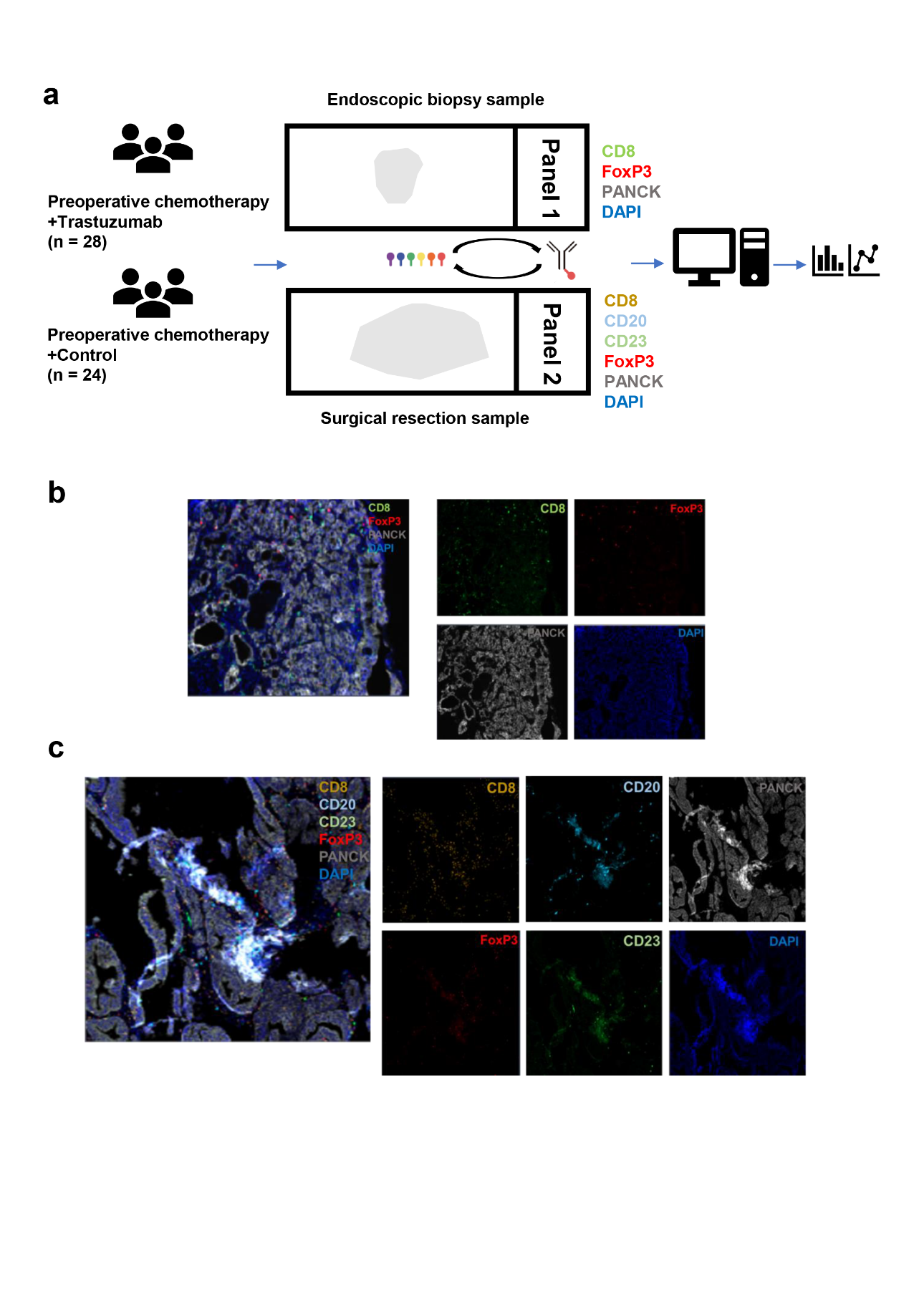


Figure S3. Multiplexed imaging assay. (a) Multiplexed immunohistochemistry workflow. A tissue section is stained and destained several times. Image analysis is performed to obtain the data. (b-c) Representative composite and single-stained images of the multiplex immunohistochemistry panels used.

# Trastuzumab

**Pre Post**


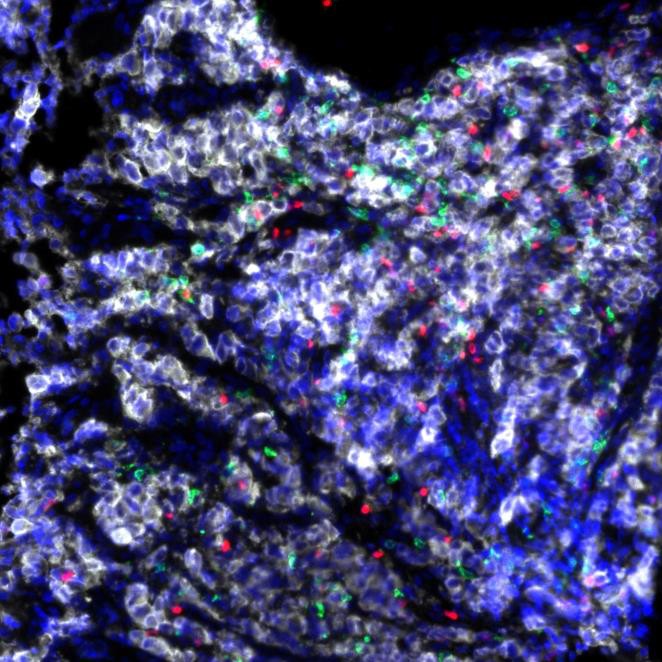


**CD8**

**FoxP3 PANCK DAPI**

100μm


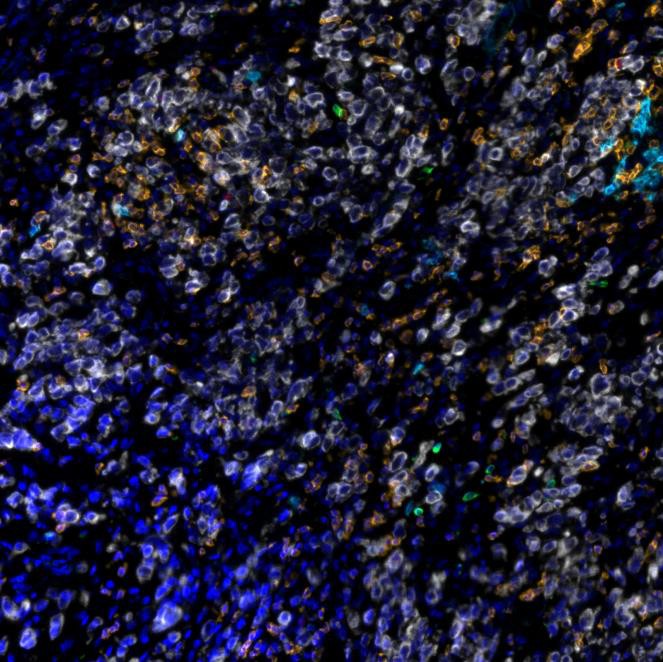


**CD8 CD20 CD23**

**FoxP3 PANCK DAPI**

100μm

# No Trastuzumab

**Pre Post**


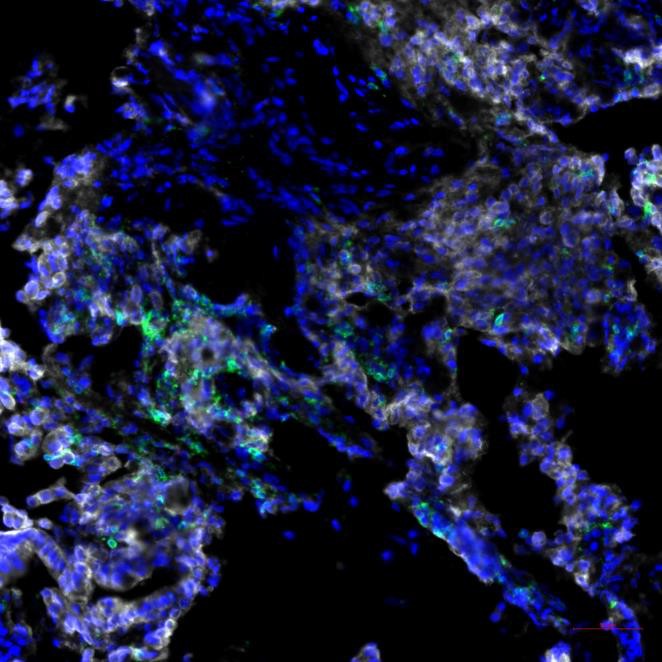


**CD8**

**FoxP3 PANCK DAPI**

100μm


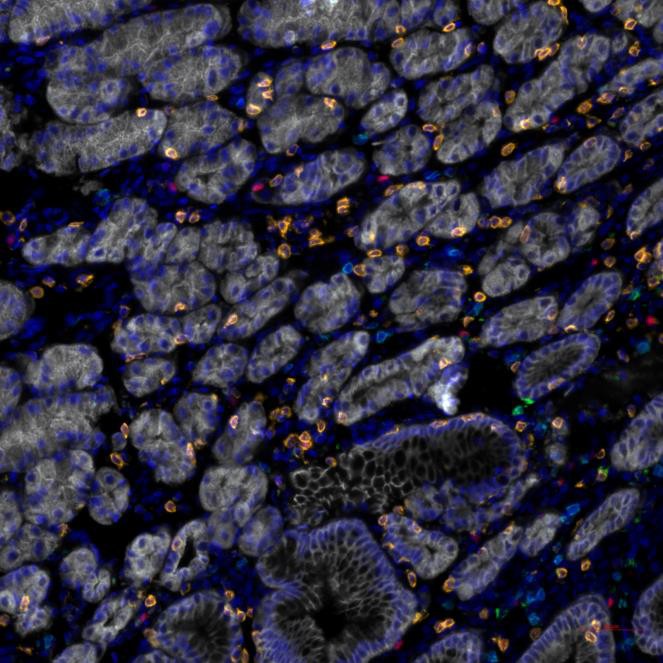


**CD8 CD20 CD23**

**FoxP3 PANCK DAPI**

100μm

Figure S4. Multiplex immunofluorescence images of immune cell

infiltration in paired pre- and post-preoperative specimens of GC.

# a

**Number of positive cells/mm2**

**1500**

**1000**

**500**

**0**

**-500**

# b

**Number of positive cells/mm2**

**1500**

**1000**

**500**

**0**

**-500**

# c

**3000**

**Number of positive cells/mm2**

**CD8-Total**


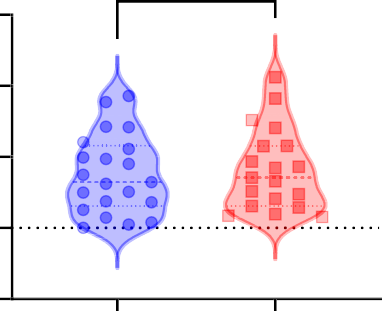
ns

**Trastuzumab No-Trastuzumab**

**CD8-Tumor**


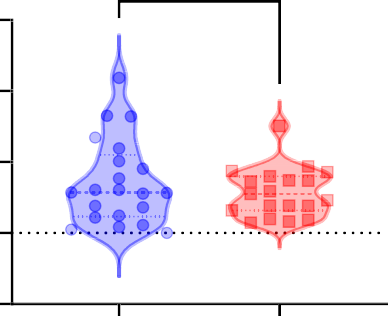
ns

**Trastuzumab No-Trastuzumab**

**CD8-Stroma**

**d**

**600**


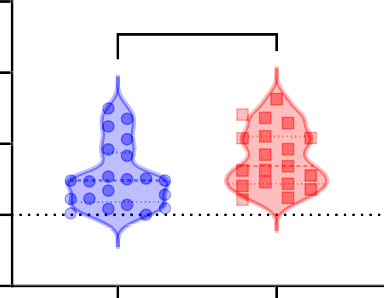


ns

**Number of positive cells/mm2**

**400**

**200**

**0**

**-200**

# e

**600**

**Number of positive cells/mm2**

**400**

**200**

**0**

**-200**

# f

**800**


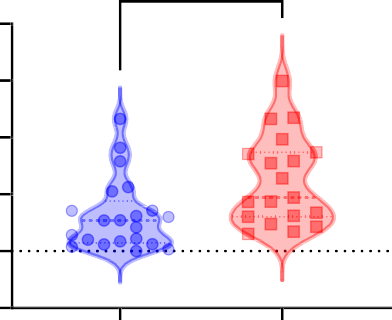


*P*=0.010

**Number of positive cells/mm2**

**FoxP3-Total**

**Trastuzumab No-Trastuzumab**

**FoxP3-Tumor**


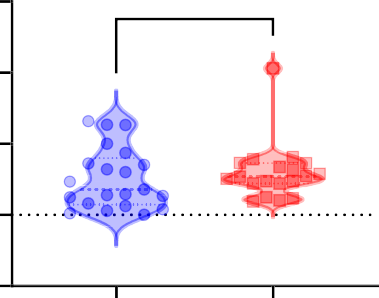
ns

**Trastuzumab No-Trastuzumab**

**FoxP3-Stroma**

✱✱

**2000**

**1000**

**0**

**600**

**400**


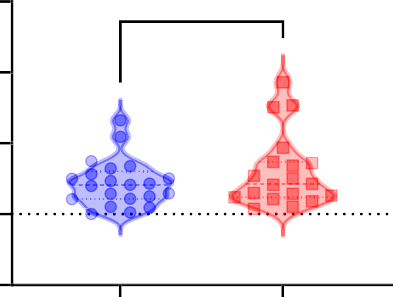


ns

**200**

**0**

**-1000**

**Trastuzumab No-Trastuzumab**

**-200**

**Trastuzumab No-Trastuzumab**

Figure S5. Immune cell infiltration in patients with HER2(+) GC before preoperative chemotherapy combined with trastuzumab

treatment or not. (a-c) Comparison of CD8+ T cell density in total (a), tumor (b) and stroma(c) before preoperative chemotherapy between two cohorts. (d-f) Comparison of FoxP3+ T cell density in total (d), tumor (e) and stroma (f) before preoperative chemotherapy between two cohorts. **p<0.01.

# a Trastuzumab


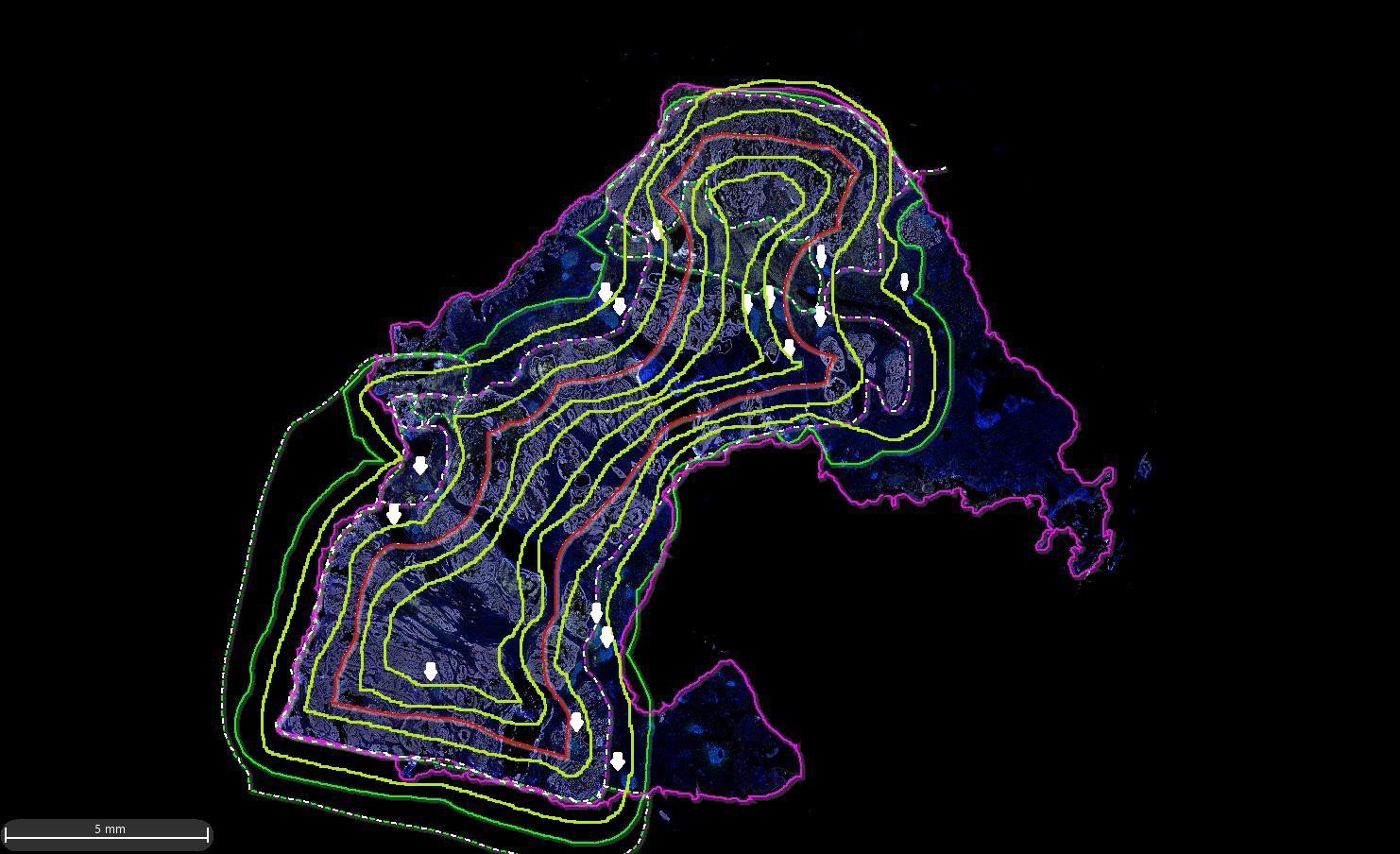


**CD8 CD20 CD23**

**FoxP3 PANCK DAPI**

5mm

**b No Trastuzumab**


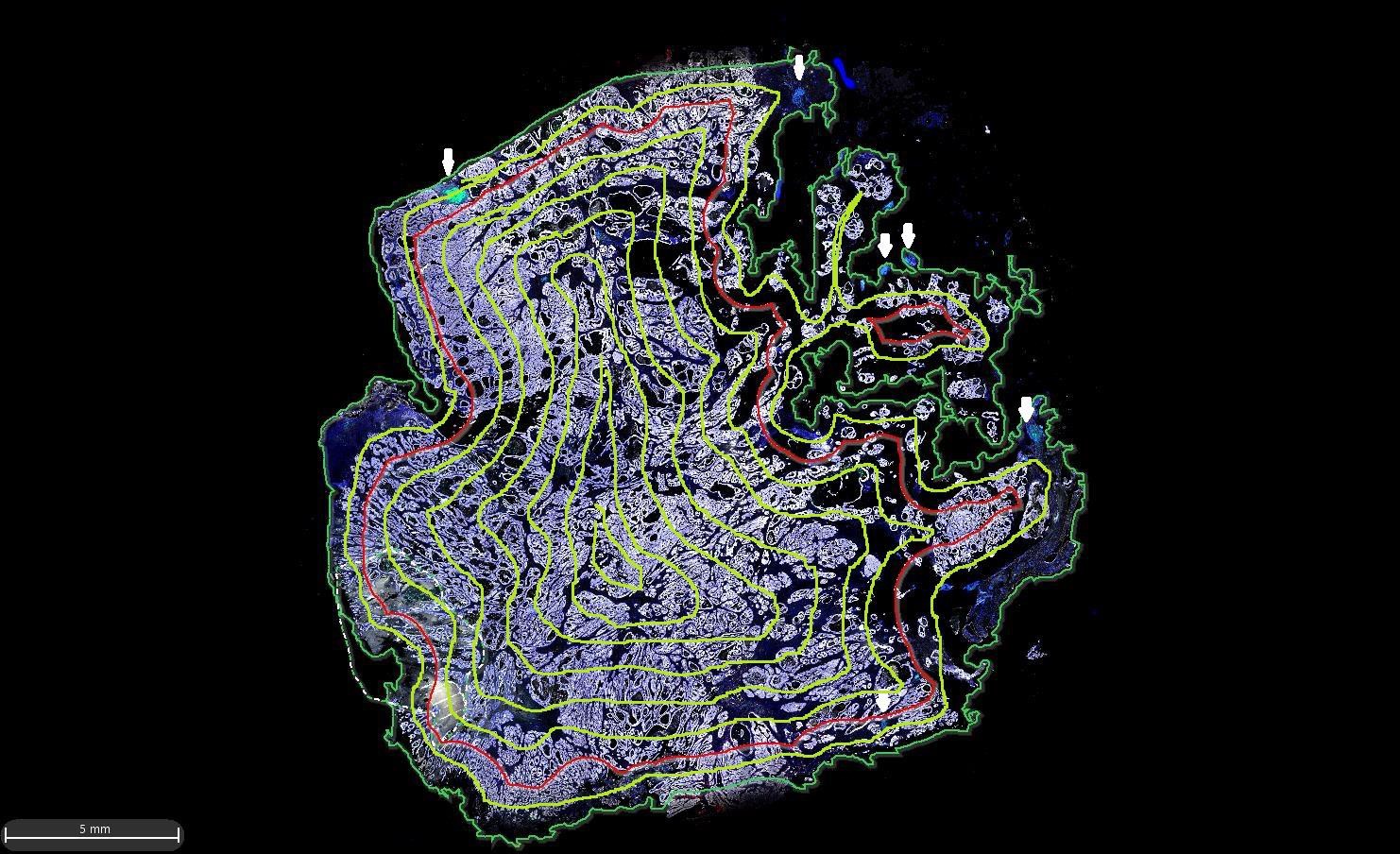


**CD8 CD20 CD23**

**FoxP3 PANCK DAPI**

5mm

Figure S6. Representative image of TLS distribution in patients with HER2(+) GC after preoperative chemotherapy combined with trastuzumab treatment or not. (a) Trastuzumab group. (b) No Trastuzumab group. The TLSs were marked by white arrows, the dividing line is green, and the distance between different regions is 500μm.
